# Lateral Heterostructure Formed by Highly Thermally Conductive Fluorinated Graphene for Efficient Device Thermal Management

**DOI:** 10.1002/advs.202401586

**Published:** 2024-04-26

**Authors:** Fanfan Wang, Zexin Liu, Jinfeng Li, Jian Huang, Li Fang, Xiaofeng Wang, Ruiwen Dai, Kangyong Li, Rong Zhang, Xiaoran Yang, Yue Yue, Zhiqiang Wang, Yuan Gao, Kai Yang, Lifu Zhang, Guoqing Xin

**Affiliations:** ^1^ Wuhan National High Magnetic Field Center and School of Materials Science & Engineering Huazhong University of Science and Technology Wuhan 430074 China; ^2^ School of Chemistry and Chemical Engineering Huazhong University of Science and Technology Wuhan 430074 China; ^3^ State Key Laboratory of Digital Manufacturing Equipment and Technology School of Mechanical Science and Engineering Huazhong University of Science and Technology Wuhan 430074 China; ^4^ School of Physics Huazhong University of Science and Technology Wuhan 430074 China; ^5^ School of Electrical and Electronic Engineering Huazhong University of Science and Technology Wuhan 430074 China; ^6^ Department of Materials Science and Engineering University of Maryland College Park MD 200742 USA

**Keywords:** current‐carrying capability, fluorinated graphene, heat dissipation, monolayer lateral heterostructure, thermal conductivity, thermal management

## Abstract

The continued miniaturization of chips demands highly thermally conductive materials and effective thermal management strategies. Particularly, the high‐field transport of the devices built with 2D materials is limited by self‐heating. Here a systematic control of heat flow in single‐side fluorinated graphene (FG) with varying degrees of fluorination is reported, revealing a superior room‐temperature thermal conductivity as high as 128 W m^−1^ K^−1^. Monolayer graphene/FG lateral heterostructures with seamless junctions are approached for device fabrication. Efficient in‐plane heat removal paths from graphene channel to side FG are created, contributing significant reduction of the channel peak temperature and improvement in the current‐carrying capability and power density. Molecular dynamics simulations indicate that the interfacial thermal conductance of the heterostructure is facilitated by the high degree of overlap in the phonon vibrational spectra. The findings offer novel design insights for efficient heat dissipation in micro‐ and nanoelectronic devices.

## Graphical abstract

Seamless graphene/fluorinated graphene lateral heterostructure is fabricated to improve heat dissipation efficiency in transistor devices. Benefiting from the highly thermally conductive and electrically insulating monolayer fluorinated graphene, efficient in‐plane heat removal paths from graphene channel to side fluorinated graphene are created. Channel peak temperature in device is significantly reduced, leading to superior current‐carrying capability.

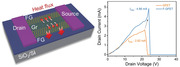

## 1 Introduction

As the transistor feature size reduces and the corresponding power density increases, the heat flux increases dramatically, forming highly localized hotspots, which seriously affects the reliability and lifespan of devices.^[^ ^1^ ^]^ As a result, efficient heat dissipation has been pursued for the continued progress of modern electronics.^[^ ^2^ ^]^ Specifically, graphene with high intrinsic carrier mobility, thermal conductivity, and mechanical flexibility shows great potential in solving this issue and has been considered a powerful competitor to silicon in the post‐Moore era.^[^ ^3^ ^]^ However, the thermal conduction of monolayer graphene attached to a substrate is constrained by the enhanced phonon‐boundary scattering facilitated by its exceedingly surface‐to‐volume ratio, impeding effective heat dissipation.^[^ ^4^ ^]^ In addition, the commonly used dielectric layers surrounding graphene, e.g., SiO_2_ and HfO_2_, present low thermal conductivities and block the heat transfer, leading to hot spots in the graphene channel.^[^ ^5^ ^]^ To address this challenge, several attempts have been carried out such as introducing functionalized molecules to enhance the heat transfer from graphene to substrate or reducing graphene channel width to enable 3D heat dissipation.^[^ ^6^ ^]^ Hexagonal‐boron nitride (h‐BN) has been recognized as the ideal substrate for 2D devices due to its atomic flat surface, excellent insulation strength, and high thermal conductivity. h‐BN has been inserted underneath graphene as heat spreader to reduce the temperature of the hot spot.^[^ ^7^ ^]^ However, the layer‐by‐layer stacking requires multiple transfer processes, which inevitably introduces impurity on the interface and degrades the device performance.^[^ ^8^ ^]^ Meanwhile, the weak van der Waals interlayer coupling induces high interfacial thermal resistance,^[^ ^9^ ^]^ impeding the efficient heat removal. A scalable, Si‐compatible and thermally favorable solution to mitigate the heat dissipation issue of graphene‐based devices is still lacking.

Fluorinated graphene (FG) has attracted extensive attentions in recent years due to its high dielectric strength, exceptional mechanical strength comparable to graphene, and robust chemical and thermal stability.^[^ ^10^ ^]^ Theoretical calculations predict that the thermal conductivity of FG can be as high as 1800 W m⁻¹ K⁻¹,^[^ ^11^ ^]^ which presents great potential in the thermal management of graphene‐based devices. FG can be obtained from graphene by controllable fluorination processes and has been applied as a passivation layer between the graphene channel and dielectric oxide substrate to reduce the scattering and improve the carrier mobility,^[^ ^12^ ^]^ demonstrating the high compatibility with graphene and other 2D materials. However, a systematical investigation of FG's thermal properties and the interfacial thermal transport between graphene and FG, which are essential to solving the overheating issue, is still missing.

In this work, elaborative investigations reveal the outstanding thermal conductivity of FG. Taking full advantage of FG's superior thermal transport capability, in‐plane graphene/FG heterojunction devices are fabricated to mitigate the self‐heating hotspot. Particularly, the thermal conductivity of the single‐sided fluorinated film has been measured as high as 128 W m^−1^ K^−1^ at room temperature, comparable to that of few‐layer BN films,^[^ ^13^ ^]^ confirming its enormous potential for thermal management. By an area‐selective patterning process, transistors with lateral graphene/FG heterojunctions have been fabricated. Compared to the graphene field‐effect transistor (GFET) where heat can only be dissipated vertically from the narrow channel into the substrate, the newly designed graphene/FG field‐effect transistor (F‐GFET) is equipped with additional heat removal paths along the in‐plane direction. Consequently, the channel peak temperature of the F‐GFET decreases by 116 K, leading to 85% and 91% improvement in the maximum power/current density, respectively. These enhancements are attributed to the outstanding thermal properties of FG and the optimal heat dissipation geometry of the lateral heterostructure device, which together establish efficient heat removal paths for graphene‐based devices. Our work provides a paradigm for the effective thermal management of graphene devices and can be extended to other 2D devices.

## 2 Results and Discussion

### 2.1 Lateral Graphene/FG Heterojunction Design for Efficient Device Cooling

**Figure 1:**
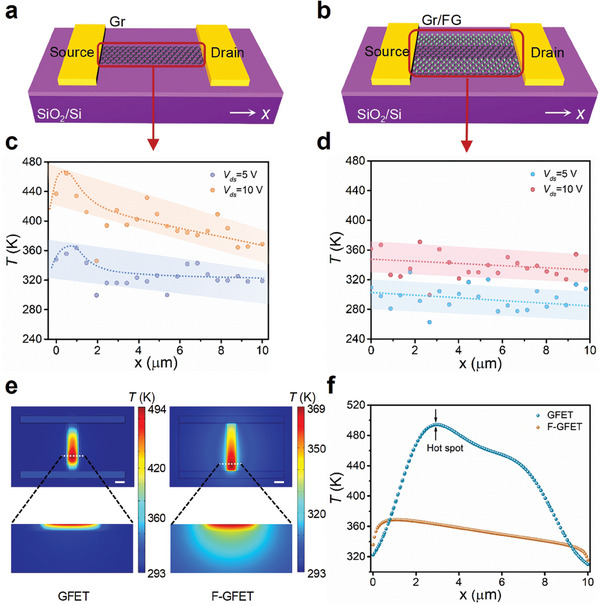
Efficient thermal management by the lateral graphene/FG heterojunction devices design. a,b) Structure schematic view of the a) GFET and b) F‐GFET. c,d) Temperature profiles along the graphene channel of the corresponding devices measured by Raman thermometry under *V*_ds_ of 5 and 10 V. e) Thermal finite‐element modeling of the GFET and F‐GFET at *V*_ds_ = 10 V, scale bar is 2 µm. The top panel shows the front view of the device, and the bottom panel shows magnified cross‐sectional view along the cutting line on the peak temperature location. f) Line profiles of temperature increases along the graphene channel.

Highly thermally conductive FG has been obtained by the fluorination of graphene and lateral graphene/FG heterojunction transistor devices have been fabricated by area‐selective patterning of graphene, as illustrated in **Figure** **1**a,b. While passing a current through the graphene channel, electrical energy is converted into Joule heat, leading to a temperature rise. The thermal profiles along the channel in the GFET and F‐GFET have been detected by micro‐Raman thermometry (Figure 1c,d; Figure S1, Supporting Information). In GFET, high thermal nonuniformities have been observed with a noticeable hotspot near the source region, resulting from a combination of a channel pinch‐off^[^ ^14^ ^]^ and current crowding.^[^ ^15^ ^]^ The overheating issue becomes more pronounced under higher applied bias (Figure 1c). In contrast, no localized overheating has been observed in F‐GFET and the peak temperature drops by 116 K at *V*_ds_ = 10 V compared to that of GFET, indicating that the FG on both sides of the channel dramatically enhances the heat removal (Figure 1d).

To study the heat dissipation of the devices, we conducted finite‐element simulations on both GFET and F‐GFET (Figure 1e,f). In the steady‐state temperature distributions (Figure 1e), a significant high‐temperature hot spot emerges near the source of GFET, while a more uniform distribution with a largely reduced peak temperature has been observed in F‐GFET. The finite‐element simulations verify the accuracy and reliability of Raman thermometry measurements. Particularly, heat spreading from graphene channel into FG can be clearly seen in the F‐GFET, leading to a significant drop of the peak temperature up to 127 K (Figure 1e,f). In GFET, heat generated in the graphene channel can only be dissipated into the underneath substrate. The small channel area limits the heat removal efficiency, resulting in a pronounced localized hotspot. In F‐GFET, heat can be transferred from graphene channel laterally into the highly thermally conductive FG, then dissipated vertically into the substrate. The heterojunction structure spreads the localized heat within the narrow channel and enlarges the heat removal paths, leading to a much lower temperature and efficiently suppressing the hotspot.

### 2.2 Controllable Synthesis and Structure Evolution of FG

**Figure 2:**
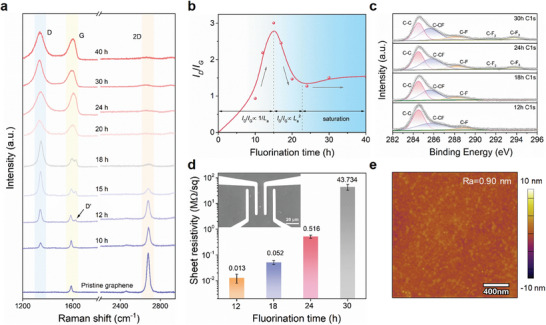
Structural and electrical characterization of FG. a) The evolution of Raman spectra of FG during fluorination. b) The *I*_D_/*I*_G_ intensity ratio as a function of fluorination time. c) C1s spectra with deconvoluted peaks of FG at different reaction times. d) The sheet resistances of FG samples versus exposure time and the inset is the optical image of a measured device for the four‐point probe technique. e) Atomic force microscopy (AFM) image of FG obtained through an H_2_ annealing followed by 24 h fluorination.

Monolayer graphene grown by chemical vapor deposition (CVD) method (details in Figure S2, Supporting Information; Experimental Section) has been first transferred to Si/SiO_2_ substrate and H_2_ annealing was carried out to clean the graphene surface. Single‐side fluorinated FG was subsequently obtained by the exposure of low‐hazard XeF_2_ gas^[^ ^16^ ^]^ (Experimental Section). The consecutive exposure results in dramatic changes in the bonding properties and structures of the film, which have been tracked by Raman spectroscopy (**Figure** **2**a). Initially, the high intensity of 2D band (2680 cm^−1^) with absence of defective D (≈1350 cm^−1^) band reflects the high crystallinity of the monolayer graphene precursor.^[^ ^17^ ^]^ After short‐time fluorination, the D band (≈1350 cm^−1^) appears, associated with the lattice disorder induced by the transition of *sp* ^2^ carbon bonds to the *sp* ^3^ C─F bonds.^[^ ^10c^ ^]^ Upon increasing the exposure time to 12 h, the defect‐activated D' band (1623 cm^−1^) appears. At longer reaction time, the D band keeps increasing, the G band merges with the D' band and continues to broaden, while the 2D band has been depressed significantly.^[^ ^18^ ^]^

The structure evolution of FG has been tracked by the *I*_D_/*I*_G_ ratios trend with exposure times (Figure 2b). It has been established that *I*_D_/*I*_G_ can be used as a footprint to measure the ratio of non‐*sp* ^2^ hybridized to *sp* ^2^ hybridized bonding states of graphene. Its correlation to the in‐plane crystallite size, *L*_a_, in graphitic materials is proportional to 1/*L*_a_ for low disorder and *L*_a_^2^ for high disorder.^[^ ^19^ ^]^ The intensity ratio of *I*_D_/*I*_G_ increases with fluorination times up to 15 h and then decreases at longer time. The initial increase of *I*_D_/*I*_G_ can be attributed to the breaking up of large crystallite inside graphene due to fluorination, where the majority of graphene film remains *sp* ^2^ crystalline phase. Further extension of fluorination time increases the portion of C─F *sp* ^3^ coverage, leading to a decrease in the number of *sp* ^2^ ordered rings and the *I*_D_/*I*_G_ ratios. We find that the *I*_D_/*I*_G_ ratios gradually stabilize after 24 h of fluorination, which indicates *L*_a_ reaches a consistent value and a turning point for the saturation of fluorination.

Following the Raman characterization, XPS has been performed to further quantify the fluorine coverage and bonding type of samples at different fluorination times. Due to the high electronegativity of fluorine, it induces strong chemical bondings in the C 1s bonding energy (Figure 2c). In addition to the notable peak of *sp* ^2^ hybridization observed at 284.5 eV and the bonding state at 288.2 eV attributed to C─F bonds, the remaining fractions of bonding states can be assigned to C─CF (285.6 eV), C─F_2_ (291.4 eV), and C─F_3_ (293.7 eV) species,^[^ ^20^ ^]^ respectively. At the early stage of fluorination within 24 h, the intensity of the C─F bond increases noticeably. When the fluorination time is further extended over 24 h, the XPS spectrum and deconvoluted peaks remain unchanged. The F/C ratio (Figure S3, Supporting Information) undergoes a process of rapid growth followed by a plateau of 24.3% at 24 h, suggesting the chemical composition of fluorinated graphene reaches stability. Based on Raman and XPS analysis, it can be concluded that the full fluorination of graphene film can be accomplished in 24 h.

Four‐probe method has been utilized to study the electrical characteristics of FG samples (Figure 2d). The square resistance of FG fluorinated by 18 h has been measured as 0.052 MΩ sq^−1^ and increases to 0.516 MΩ sq^−1^ after a 24 h XeF_2_ exposure, indicating that the conductive graphene can be turned into a highly insulating state by fluorination. The fluorination reduces the charge on the conductive *π* orbitals, introduces scattering centers, and opens bandgaps, leading to high resistance.^[^ ^10c^ ^]^ However, when the fluorination time is further extended to 30 h, the square resistance sharply increases to 43.734 MΩ sq^−1^. At longer fluorination time, high‐density cracks have been observed (Figure S4, Supporting Information) and the FG film has been broken down into small pieces, leading to a dramatic increase of resistivity. These findings align well with the results obtained from XPS and Raman testing, further proofing that 24 h is an optimal fluorination time. Meanwhile, fully fluorinated samples present a long‐term stability of in the atmosphere, favorable for the wide applications (Figure S5, Supporting Information).

It is worth noting that the H_2_ annealing process before the fluorination is effective in obtaining clean FG films. Figure 2e presents the AFM image of FG surface obtained by an H_2_ annealing and fluorination process. The FG surface appears remarkably clean and smooth, with a low surface roughness (*R*_a_) of 0.9 nm. As a comparison, FG films synthesized with Ar annealing or without annealing (Figure S6, Supporting Information) show large particles and wrinkles on the surface. The organic residues on the graphene surface originating from the PMMA during the transfer process can be fluorinated in XeF_2_ gas, leaving unremovable contamination on the FG surface. H_2_ annealing decomposes the organic residues, thus achieving a clean surface and providing high‐quality film for device fabrication.

### 2.3 Measurement and Modulation of FG's Thermal Conductivity

**Figure 3:**
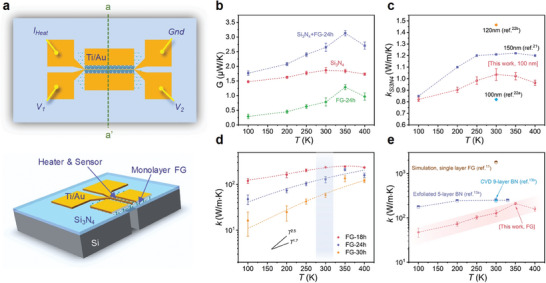
Electrical thermometry platform and measured thermal properties of FG. a) Top and cross‐sectional view of the schematic of suspended electrical thermometry platform. b) Thermal conductance of individual FG film, supporting Si_3_N_4_ membrane and the combination. c) Thermal conductivities of Si_3_N_4_ membrane on the platform and the comparison to previously reported Si_3_N_4_ films with different thicknesses.^[^ ^21^, ^22^ ^]^ d) Temperature‐dependent thermal conductivities of FG with various fluorination times. FG‐18 h, FG‐24 h, and FG‐30 h stand for samples fluoridated for 18, 24, and 30 h, respectively. e) Temperature‐dependent thermal conductivities of our supported FG samples. Shown in comparison are the reported experimentally measured thermal conductivities of exfoliated five‐layer suspended BN,^[^ ^13a^ ^]^ CVD‐grown 9‐layer suspended BN,^[^ ^13b^ ^]^ as well as the theoretically calculated thermal conductivity of monolayer FG.^[^ ^11^ ^]^

A modified suspended thermometry bridge has been applied for the thermal conductivity measurements (**Figure** **3**a)^[^ ^21^ ^]^ (details in Figure S7, Supporting Information; Experimental Section). Thin suspended Si_3_N_4_ film on a Si trench has been used to provide thermal isolation and mechanical stability, and single‐layered FG has been transferred on the top of Si_3_N_4_ film. A metal line deposited on the FG surface acts as both heater and temperature sensor. We perform heat flow measurements from 100 to 400 K on FG samples of different exposure times under a vacuum environment where heat loss due to convection and radiation is negligible. During measurement, a heating power *P*_H_ is applied to the heater electrode, and the temperature rise is detected by monitoring the change in the calibrated resistance of the metal line. This allows us to obtain the variation of the temperature Δ*T*_S_ as a function of the heating power *P*_H_ (see Experimental Section; Figure S8, Supporting Information). Owing to the thick Si substrate and deposited large area Au outside the window acting as heat sinks, the FG temperature at the edge of the Si trench always drops to the ambient temperature. Thus, Δ*T*_S_ represents the temperature change from the heater to the window edge. The thermal conductance of FG layer (*G'*) can be extracted by subtracting the pure Si_3_N_4_ film thermal conductance (*G*_Si3N4_) from the total thermal conductance (*G*_T_) of the FG stacked with supporting Si_3_N_4_ film (Figure 3b).

Since the heat flow is symmetric on the two sides of the line heater, we consider 50% of the *P_H_* has been transferred into each side of Au heat sink. We find that although the 1D heat transfer problem due to the geometric size of the suspended film has been considered during the measurement, 3.6% of the heat inevitably gets lost at the end of the trench as verified through finite element simulations (Figure S9, Supporting Information; Note S1, Supporting Information). Therefore, after the incorporation of heat loss into our analytical model, the total thermal conductance and thermal conductivity (*k*) of FG can then be obtained as:

(1) $$G_{T}=\frac{P_{H}\left(1-\beta \right)}{2\Delta T_{S}}$$

(2) $$k=G^{\prime }\frac{L}{Wh}$$

where *β* denotes the heat loss along the parallel direction to the Si substrate, *L* is the distance between the heater and the window boundary, and *W* and *h* are the width and thickness of FG, respectively. For method verification, we find the thermal conductivity of our Si_3_N_4_ layer ranges from 0.83 to 1.04 W m^−1^ K^−1^ at the temperature from 100 to 300 K, matching well with the data in the literature^[^ ^21^, ^22^ ^]^ (Figure 3c). We also conducted thermal conductivity measurements of monolayer graphene using the same platform, as shown in Figure S10 (Supporting Information). Within the temperature range of 100 K to 400 K, the thermal conductivity of monolayer graphene varies from 184 to 390 W m^−1^ K^−1^. The measured thermal conductivities closely align with the data reported in the literature for supported graphene,^[^ ^21^ ^]^ providing strong confidence in the reliability of our testing methods.

The thermal conductivities of FG with different fluorination times as a function of temperature have been extracted (Figure 3d). The thermal conductivity of FG film gradually increases with temperature, reaching a maximum at around 350 K. Over the low *T* region of 100–300 K, the thermal conductivities of the monolayer FG follow a power law temperature trend between *T* ^1.7^ and *T* ^2.5^, similar to that of supported single‐layer graphene and graphene nanoribbons.^[^ ^3^, ^4^, ^23^ ^]^ The extension of fluorination time leads to a decreasing trend in thermal conductivity, which is consistent with the increase of structural defects observed from Raman and SEM (Figure 2; Figure S4, Supporting Information).

Remarkably, our insulating FG films exhibit exceptional thermal conductivities (Figure 3e), for instance, the room temperature thermal conductivity of FG fluorinated at 18 and 24 h reaches 236 ± 5 and 128 ± 20 W m^−1^ K^−1^, respectively. It is worth noting that the thermal conductivity of supported monolayer FG is much lower than the theoretically calculated value.^[^ ^11^ ^]^ This may be attributed to the strong interactions between FG and the substrate, as well as the presence of grain boundaries,^[^ ^24^ ^]^ and the defects from fluorination, together enhance the phonon scattering. The measured thermal conductivities are compared to that of exfoliated and CVD‐grown few‐layer h‐BN, which has been one of the few discovered thermally conductive and electrically insulating 2D materials. Our synthesized FG can be the new member of the insulating 2D material family, and the excellent thermal conductivity endorses its huge potential for heat dissipation in 2D electronic devices.

### 2.4 Long‐Range Ordered Atomic Structure of FG and the Seamless Graphene/FG Heterostructure

**Figure 4:**
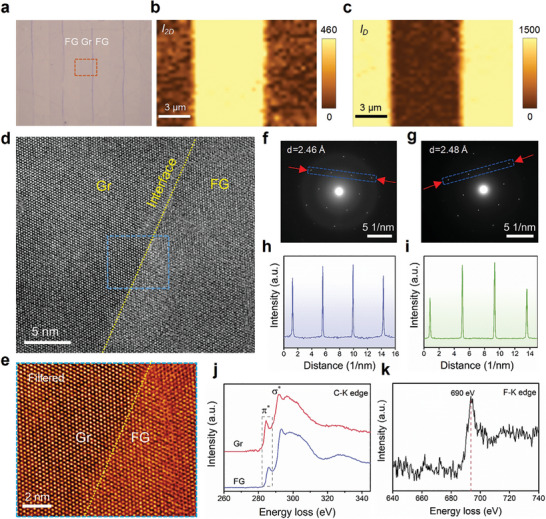
The long‐range order of FG and the seamless graphene/FG lateral heterostructure. a) Optical micrography image of alternating stripes of graphene and FG. b,c) Raman mapping of the 2D (b) and D (c) bonds from the dashed box region shown in (a). d) Atomic‐resolution TEM image of graphene/FG heterostructure interface, highlighted by the yellow dashed line. e) Filtered TEM image of the square area in (d). f,g) Selected area electron diffraction (SAED) patterns obtained for graphene and FG. h,i) Corresponding intensity line profiles along with the blue rectangle in (f,g), respectively. j,k) Electron energy loss spectroscopy (EELS) data of j) carbon K‐edge measured for two phases and k) fluorine K‐edge corresponding to FG.

Graphene/FG heterostructures have been approached by an area‐selective patterning process (see Experimental Section for details) in order to broaden the application of highly thermally conductive FG in nanoelectronics cooling. **Figure** **4**a presents the optical image of alternating stripes of graphene/FG with noticeable optical contrast. Raman mapping has been applied to elaborately characterize the graphene/FG heterojunction and the characteristic D and 2D bands have been employed to identify the FG and graphene region, respectively (Figure 4b,c). Graphene and FG are bonded seamlessly, which can be attributed to the in‐situ fluorination process that allows the strong chemical bonding between graphene and FG and facilitates the heat transfer between each other. Sharp boundaries with distinct spatial distribution of both D and 2D Raman signals indicate that the controllable fluorination process allows the formation of well‐defined conductive and insulating regions on a single atomic thick sheet.

The long‐range atomic order of FG has been confirmed by the systematic characterization of atomic structure and composition via high‐resolution TEM and SAED (Figure 4d–k). Similar to graphene, hexagonal lattices covering a large area have been observed in the FG region (Figure 4d,e). In addition, the SAED patterns of both graphene and FG region show sixfold symmetry with sharp intensity, indicating a high degree of order (Figure 4f,g). The higher intensity of the first‐order diffraction spots compared to the second‐order spots in both graphene and FG suggests that the fluorination process well preserves the continuous monolayer structure without crumpling and folding of the layer^[^ ^16b^ ^]^ (Figure 4h,i). The EELS shows that the π* peak (285 eV) on the carbon K‐edge spectrum has been strongly suppressed in the FG region, and a new peak of 690 eV shows up on the fluorine K‐edge spectrum,^[^ ^25^ ^]^ further confirming the coverage of C─F bonds and the graphene/FG heterostructure. The substrate‐supported graphene limits the fluorination to the top surface, avoiding the high‐density lattice disruption and amorphization due to the local over‐fluorination seen in the two‐side fluorination.^[^ ^26^ ^]^ The highly ordered 2D lattice structure of FG in the long range has been well retained from the pristine graphene after the fluorination, contributing to the superior thermal conductivity.

The seamless interface of graphene/FG heterostructure has been confirmed by the structure analysis. At the interface, it can be seen that chemical bonding has been well‐preserved on the atomic scale between the graphene/FG during the fluorination (Figure 4d,e). The lattice constant of FG (2.48 Å) obtained from the SAED patterns (Figure 4g,f) is consistent with previous reports^[^ ^16a^ ^]^ and shows a low mismatch (0.81%) with that of graphene (2.46 Å). The neglectable lattice expansion allows the smooth transition of the *sp* ^2^ C─C bonds on the graphene side to *sp* ^3^ configuration on the FG side, without lattice distortion and vacancy seen along the interface line. The strong chemical bonding ensures a low interfacial thermal resistance, which is essential for lateral heat transfer.

### 2.5 Electrical Performance and Heat Dissipation Improvement of the F‐GFET

**Figure 5:**
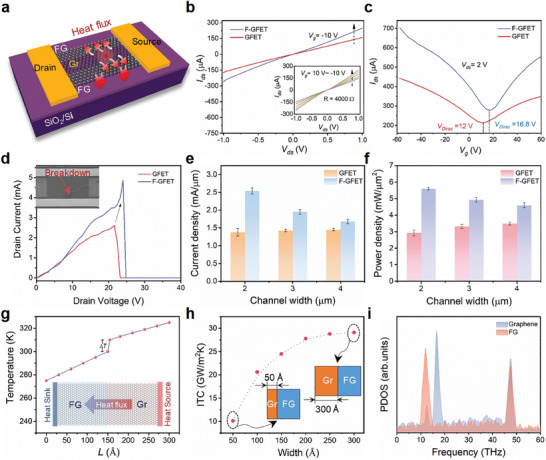
Electrical measurement of graphene/FG lateral heterostructure device and MD simulation of interfacial thermal dissipation. a) Schematic diagram of a F‐GFET device with additional lateral heat removal paths. b) Output characteristics (*I*_ds_‐*V*_ds_) of the GFET and the F‐GFET with the same graphene dimensions. The inset shows the output curves as a function of gate bias in the F‐GFET device. c) Transfer curves (*I*_ds_–*V*_g_) for the GFET and F‐GFET, where the drain‐source voltage is kept at 2 V. d) High field breakdown of the devices with 2 µm in channel width, where the top inset shows the SEM image of the measured F‐GFET after electrical breakdown. e) Maximum current density and f) power density as a function of graphene channel width for GFET and F‐GFET devices. The width of FG in the heterojunction devices remains constant at 10 µm. g) The steady‐state temperature profile of the graphene/FG lateral heterostructure in MD simulation. The inset is the schematic diagram of the simulation model. h) Interfacial thermal conductance (ITC) plotted with graphene width, where the FG width is kept constant at 300 Å. i) Phonon density of states (PDOS) for graphene and FG at the lateral heterostructure interface.

Transistors have been built up based on the graphene/FG lateral heterostructure (**Figure** **5**a–c), showing p‐doped behaviors with great improvement in carrier mobility. The linear output characteristic of GFET and F‐GFET indicates perfect Ohmic contacts and higher *I*_ds_ have been observed for the F‐GFET (Figure 5b). The mobility of F‐GFET has been extracted as 1650 cm^2^ V^−1^ s^−1^, much higher than that of GFET (992 cm^2^ V^−1^ s^−1^). The graphene/FG lateral heterostructure allows graphene edges to terminate with *sp* ^3^ carbon covalently bound to fluorine, rather than a combination of dangling bonds, *sp* ^2^ and *sp* ^3^ hybridized carbon bound to a variety of oxygen‐rich functional groups left by O_2_ plasma etching. The well‐defined edge termination reduces boundary scattering,^[^ ^27^ ^]^ thus favoring the carrier mobility. The transfer characteristic curves (*I*_ds_‐*V*_g_) with applied *V*_ds_ of 2 V are shown in Figure 5c. As we can see, F‐GFET shifts the neutrality point of GFET from 12 to 16.8 V, which can be attributed to the side FG acting as electron acceptors and strongly p‐doping graphene.^[^ ^28^ ^]^

The implementation of graphene/FG heterostructure enhances the heat removal of graphene devices and its current‐carrying capability significantly by providing facile cooling paths. Electrical breakdown behaviors of GFET and F‐GFET were studied under a high in‐plane electric field at zero gate voltage, as shown in Figure 5d. When the drain voltage was swept from 0 to 40 V, the breakdown current of F‐GFET with 2 µm channel width was measured up to 4.8 mA, approximately two times as high as that of GFET. Statistically, the maximum current and power density of the F‐GFET (2 µm channel width) reaches 2.54 ± 0.08 mA µm^−1^ and 5.60 ± 0.07 mW µm^−2^, representing 85% and 91% improvement compared to that of GFET (Figure 5e,f). In the failed device (Figure 5d), a crack has been observed near the source contact, confirming the formation of hotspots within the channel and matching with previous Raman thermometry and finite element simulations. The dramatic improvement in the current‐carrying capability of F‐GFET can be attributed to the excellent thermal conductivity of FG and the seamless lateral heterojunction, enabling efficient heat spreading and dissipation paths to eliminate the self‐heating of the graphene channel.

Following this, we further studied the maximum current density and power density of GFET and F‐GFET with different channel widths when subjected to current breakdown (Figure 5e,f). We note that for GFET, their maximum current/power at breakdown shows weak dependence with respect to the channel width. The slight increases in current/power density at a larger width can be related to the reduced proportion of defective edges, which behave as weak points at high temperatures.^[^ ^29^ ^]^ In contrast, for F‐GFET, noticeable improvements in the maximum current/power density have been seen in the narrower devices. Steady‐state finite element simulations have been carried out to confirm that when the channel width reduces from 4 to 2 µm, the proportion of the in‐plane heat flux from graphene to FG over the total Joule heating increases from 13.01% to 26.78%, consequently boosting the carrying capacity of the device (see Figure S11, Supporting Information; Note S2, Supporting Information). In the wide channel devices, the majority of the heat has been directly transferred from the graphene region vertically into the underneath substrate. Thus, only minor improvement in the electrical‐thermal performance has been observed after the incorporation of FG. In contrast, the narrow channel device with a shrunk area limits the vertical heat transfer, and a larger proportion of heat can be conducted laterally into FG and then dissipated into the substrate, which effectively prevents premature Joule breakdown of the device. Therefore, such a lateral heterostructure is particularly more favorable for the thermal management of smaller‐sized devices.

To obtain a deeper insight into the phonon mechanisms of lateral thermal transport from graphene to FG, we conducted non‐equilibrium molecular dynamics (NEMD) simulations on the graphene/FG planar heterostructure (Figure 5g inset). A temperature difference of ≈50 K was artificially introduced to initiate the heat transfer from graphene to FG. After the system reaches a nonequilibrium steady state, a distinct temperature jump occurs at the interface (Figure 5g), suggesting the existence of interfacial thermal resistance between graphene and FG. With an increase in the width of graphene, the ITC of the system is enhanced from 10.1 × 10^9^ W K^−1^ m^−2^ to a saturated value of 29.1 × 10^9^ W K^−1^ m^−2^ (Figure 5h), which is approximately four orders of magnitude higher than that of van der Waals interfaces^[^ ^8^ ^]^ and nearly two times higher than the in‐plane thermal conductance between graphene and *h*‐BN (10.21 × 10^9^ W K^−1^ m^−2^).^[^ ^30^ ^]^ In addition, a larger width can reduce the phonon‐boundary scattering of phonons with larger phonon mean free paths, which can be transmitted across the interface with lower inelastic scattering. Therefore, the ITC value is increased by a larger width, as shown in Figure 5h. The PDOS for graphene and FG within the planar heterostructure was calculated to understand the phonon mechanisms, as presented in Figure 5i. The fluorination of graphene causes a red shift of the low‐frequency phonons (10–15 THz). The low‐frequency phonons correspond to the flexural motion,^[^ ^31^ ^]^ which is hindered by the mass increase (grafting fluorine atom) and the transition of carbon bond hybridization from in‐plane *sp* ^2^ to out‐of‐plane *sp* ^3^. Nevertheless, the phonon spectra of the graphene and FG possess a high degree of overlap in the high‐frequency range of 45–50 THz, which corresponds to the in‐plane motion and reflects the high quality of in‐plane phonon transport at the interface. This result further implies that in‐plane phonons are the main contributors to the interfacial heat thermal transport across the interface. The atomistic simulation has confirmed the high feasibility of the graphene/FG heterostructure for the thermal management of graphene devices.

## 3 Conclusion

In summary, highly thermally conductive FG has been approached and monolayer graphene/FG lateral heterostructures have been fabricated to improve the device current‐carrying capability by better manipulating the heat dissipation and reducing the channel temperature. The long‐range ordered structure provides single‐sided fluorinated FG a thermal conductivity as high as 128 W m^−1^ K^−1^ at room temperature with excellent electrical insulation. Seamless graphene/FG lateral heterostructures together with the well‐matched PDOS spectrum ensure the high ITC. Consequently, the graphene/FG lateral heterostructure significantly facilitates the Joule heat removal by offering additional heat removal path along the in‐plane direction. The channel temperature has been dramatically lowered and current‐carrying capability has been improved. Particularly, the lateral heterostructure is demonstrated more favorable for the cooling of smaller‐sized devices. Our results provide a paradigm for the thermal management of emerging micro‐ and nanoscale electronic devices.

## 4 Experimental Section

### Growth and Transfer of Monolayer Graphene

Single‐layered graphene was obtained by using atmospheric pressure CVD (APCVD). The commercially available Cu foil (25 µm thickness, 99.9% purity, XFNANO) was first electrochemically polished to improve its surface roughness. The electrolytes consisted of phosphoric acid and glycol (v:v = 3:1). A second Cu foil served as the anode and 2 V voltage was applied for about 30 min. After cleaning, the Cu foil was loaded into the central of the furnace tube and annealed at 1030°C under 500 sccm Ar and 10 sccm H_2_ for 60 min. 5–30 sccm CH_4_ (0.1% in Ar) was then introduced for about 60 min during the graphene growth. After growth, CH_4_ gas was switched off, and the furnace was quickly cooled down to room temperature. The as‐grown monolayer graphene film was transferred to target substrates by using conventional poly(methyl methacrylate) (PMMA)‐assisted method for subsequent characterization and electrical device fabrication.^[^ ^32^ ^]^

### Fluorination of Graphene

The CVD‐grown graphene films were first transferred to SiO_2_/Si substrate, followed by annealing up to 300 °C under an H_2_–Ar (10% H_2_) mixed flow for 2 h. Then, graphene films were fluorinated in XeF_2_ gas at 70 °C with controlled time of 10 h to 40 h to fabricate samples with various degrees of fluorination. The fluorination process was carried out under an inert atmosphere to avoid deliquescence.

### Synthesis of Graphene/FG Heterostructures

The graphene/FG heterostructures were fabricated according to the following procedure: i) CVD‐grown monolayer graphene was transferred onto SiO_2_/Si substrate labeled with number markers and patterned into rectangles by maskless lithography followed by an O_2_ plasma etching step, ii) the graphene channel was precisely determined in the middle of the rectangular with the help of the markers, and then 20 nm thick Al film was deposited using an e‐beam evaporator to obtain Al strip mask. The devices had a channel width of 2 µm with a channel length of 10 µm, iii) after H_2_ annealing, the rest of the graphene rectangular was fluorinated by exposure to XeF_2_ gas for 24 h, followed by etching of the Al mask. The graphene/FG lateral heterostructure was completed after that.

### Fabrication and Measurement of Thermal Conductivity Platform

The valid thermal conductivities of the monolayer pristine graphene and FG were probed using the suspended electrical thermometry method. The measurement platforms were constructed according to the following procedures. Briefly, graphene or FG films were first transferred to cover over the Si_3_N_4_ membrane (100 nm thickness) window on top of silicon supporting frames, obtained from YW MEMS (Suzhou) in this study. Subsequently, standard photolithography processes were applied to pattern the metal electrodes. Ti (10 nm)/Au (40 nm) films were deposited to form heater/sensor electrodes as well as the metal heat sink and contact pads. Note that for the graphene sample, 20 nm of Al_2_O_3_ was deposited by atomic layer deposition prior to evaporating the electrodes, allowing for electrical insulation.

As‐prepared suspended electrical thermometry devices were placed in a lakeshore TTPX probe station under vacuum of 10^−6^ Torr. The ambient temperature was controlled by a lakeshore 336 temperature controller and liquid nitrogen cooling system. The electrical measurements were carried out using the Keithley 6221/2182A ultra‐low resistance test system. During measurements, the temperature coefficient of resistance (TCR) for the heater was first calibrated by a 4‐point Delta mode. Then, heating power (5 to 150 µW) was applied to the heater, meanwhile, the corresponding temperature rises were monitored by a 4‐point current–voltage test. Thus, the thermal conductance of the sample can be obtained based on the temperature rise of the central electrode, the applied heating power, and the geometry of the sample.

### Device Fabrication and Measurements

Graphene/FG heterostructures were applied as channel materials to fabricate electrical devices. Cr/Au (15/35 nm) was deposited on the two ends of the heterostructure sample to form source and drain electrodes. The electrical measurements of the devices were performed by a Keithley 2636B at room temperature. The field‐effect carrier mobility from the transfer characteristics was estimated based on the following equation:^[^ ^33^ ^]^

(3) $$\mu =\left(\frac{dI_{\mathrm{ds}}}{dV_{g}}\right)\left(\frac{L}{WC_{\mathrm{OX}}V_{\mathrm{ds}}}\right)$$

where *L* and *W* are the channel length and width, respectively. *C*_ox_ (*C*_OX_ = ε_r_ε_0_/*d* ) is the gate capacitance (≈12 nF cm^−2^ for 285 nm thick SiO_2_) and d*I*_ds_/d*V*_g_ is the slope of the linear regions of the transfer curves.

### Measurement of Device Channel Temperature Profile

In addition to electrical analysis, the temperature profiles along the device channel in GFET and F‐GFET have been measured by Raman thermometry method.^[^ ^15b^ ^]^ The prepared devices were placed on a Linkam THMS600 heating stage, where the temperature was regulated using a heating system controller and liquid nitrogen cooling, while external connections to the source meter provided the electrical bias. First, Raman peak shifts versus temperature for graphene were calibrated on the hot stage. Then, temperature profiles were collected by measuring the Raman peak shifts across the channel in the biased devices. During the calibration and measurement, low‐power laser was applied so that the temperature rise due to laser heating could be neglected.

### MD Simulation

Nonequilibrium molecular dynamics simulations (NEMD) were performed to study the thermal transport across the graphene/FG interface using the LAMMPS software package.^[^ ^34^ ^]^ The Tersoff potential was used to characterize the interactions between C and F atoms. Fixed boundary conditions were applied in the out‐of‐plane direction and the in‐plane thermal transport direction, while the periodic boundary condition was applied in the other in‐plane direction that is perpendicular to the thermal conduction. The atoms at the two ends of the heat flux direction were held fixed. After initial energy minimization, the system was relaxed for 500 ps in the isothermal‐isobaric (NPT) ensemble, maintaining a temperature of 300 K and a pressure of 1 atm. The time step was set as 0.1 fs. Then, the system was further relaxed in the canonical (NVT) ensemble under the same temperature for another 500 ps. Next, the system was switched to the microcanonical (NVE) ensemble, with the temperatures of the heat source and heat sink maintained at 330 and 275 K, respectively, by two separate Langevin thermostats. After 3 ns, a stable temperature profile in the heat conduction direction was established. We then calculated the ITC of the system according to:

(4) $$\lambda =\frac{J}{\Delta T}$$

where *λ* represents the ITC, △*T* is the temperature difference at the interface, and *J* is the heat flux across the interface. Furthermore, in the analysis of phonon modes, to quantify the overlap of phonon spectra, the overlap value (*S*) is defined as:

(5) $$S=\frac{\int D_{c}\left(\omega \right)D_{F}\left(\omega \right)d\omega }{\int D_{c}\left(\omega \right)d\omega \int D_{F}\left(\omega \right)d\omega }$$

where *D_C_* (*ω*) and *D_F_* (*ω*) are the normalized PDOS of the C atoms and F atoms at frequency *ω*, respectively. The PDOS was calculated via:^[^ ^35^ ^]^

(6) $$\mathrm{PDOS}\left(\omega \right)=\int_{-\infty }^{+\infty }e^{i\omega t}\mathrm{VACF}\left(t\right)dt$$

where VACF represents the velocity autocorrelation function, defined as:

(7) $$\mathrm{VACF}\left(\tau \right)=\frac{1}{N}\sum_{i=1}^{N}\langle v_{i}\left(0\right)\cdot v_{i}\left(\tau \right)\rangle$$

In this expression, *N* represents the total number of atoms in the computational domain, and *v_i_* (0) and *v_i_* (*t*) denote the velocity vector of the *i* ^th^ atom at *t*  =  0 and τ, respectively. The bracket stands for the ensemble average.

### Characterizations Instruments

The morphology and structure of graphene and FG were taken with optical microscopy, Raman (WiTec Alpha 300, 532 nm laser), scanning electron microscope (SEM, Thermo Scientific Scios 2), and transmission electron microscope (TEM, FEI Talos F200X). EELS characterizations were performed using a double spherical aberration‐corrected TEM (JEOL JEM‐ARM200CF, at 80 kV). The AFM images were collected using the Shimadzu SPM9700 system. X‐ray photoelectron spectra(XPS, Kratos AXIS SUPRA+) were measured to determine the ratio of fluorine to carbon atoms in FG.

## Conflict of Interest

The authors declare no conflict of interest.

## Supporting information

Supporting Information

## Data Availability

The data that support the findings of this study are available from the corresponding author upon reasonable request.

## References

[1] H. N. Khan , D. A. Hounshell , E. R. Fuchs , Nat. Electron. 2018, 1, 14.

[2] a) M. Qin , Y. Xu , R. Cao , W. Feng , L. Chen , Adv. Funct. Mater. 2018, 28, 1805053;

[3] a) T. Ma , Z. Liu , J. Wen , Y. Gao , X. Ren , H. Chen , C. Jin , X. L. Ma , N. Xu , H. M. Cheng , Nat. Commun. 2017, 8, 14486; 28205514 10.1038/ncomms14486 PMC5316893

[4] J. H. Seol , I. Jo , A. L. Moore , L. Lindsay , Z. H. Aitken , M. T. Pettes , X. Li , Z. Yao , R. Huang , D. Broido , Science. 2010, 328, 213. 20378814 10.1126/science.1184014

[5] a) M. H. Bae , Z. Y. Ong , D. Estrada , E. Pop , Nano Lett. 2010, 10, 4787; 20521804 10.1021/nl1011596

[6] a) H. Han , Y. Zhang , N. Wang , M. K. Samani , Y. Ni , Z. Y. Mijbil , M. Edwards , S. Xiong , K. Sääskilahti , M. Murugesan , Nat. Commun. 2016, 7, 11281; 27125636 10.1038/ncomms11281 PMC4855536

[7] D. Choi , N. Poudel , S. Park , D. Akinwande , S. B. Cronin , K. Watanabe , T. Taniguchi , Z. Yao , L. Shi , ACS Appl. Mater. Interfaces. 2018, 10, 11101. 29528211 10.1021/acsami.7b16634

[8] C.‐C. Chen , Z. Li , L. Shi , S. B. Cronin , Appl. Phys. Lett. 2014, 104, 081908.

[9] C. Wang , J. Guo , L. Dong , A. Aiyiti , X. Xu , B. Li , Sci. Rep. 2016, 6, 25334. 27142571 10.1038/srep25334 PMC4855177

[10] a) W. Feng , P. Long , Y. Feng , Y. Li , Adv. Sci. 2016, 3, 1500413; 10.1002/advs.201500413 PMC5115570 27981018

[11] W. Huang , Q. X. Pei , Z. Liu , Y. W. Zhang , Chem. Phys. Lett. 2012, 552, 97.

[12] K. I. Ho , M. Boutchich , C. Y. Su , R. Moreddu , E. S. R. Marianathan , L. Montes , C. S. Lai , Adv. Mater. 2015, 27, 6519. 26398725 10.1002/adma.201502544

[13] a) I. Jo , M. T. Pettes , J. Kim , K. Watanabe , T. Taniguchi , Z. Yao , L. Shi , Nano Lett. 2013, 13, 550; 23346863 10.1021/nl304060g

[14] a) W. A. Shockley , Proc. IEEE. 1952, 40, 1365;

[15] a) K. L. Grosse , M. H. Bae , F. Lian , E. Pop , W. P. King , Nat. Nanotechnol. 2011, 6, 287; 21460825 10.1038/nnano.2011.39

[16] a) R. R. Nair , W. Ren , R. Jalil , I. Riaz , V. G. Kravets , L. Britnell , P. Blake , F. Schedin , A. S. Mayorov , S. Yuan , Small. 2010, 6, 2877; 21053339 10.1002/smll.201001555

[17] J. E. Lee , G. Ahn , J. Shim , Y. S. Lee , S. Ryu , Nat. Commun. 2012, 3, 1024. 22929781 10.1038/ncomms2022

[18] A. C. Ferrari , Solid State Commun. 2007, 143, 47.

[19] K. I. Ho , J. H. Liao , C. H. Huang , C. L. Hsu , W. Zhang , A. Y. Lu , L. J. Li , C. S. Lai , C. Y. Su , Small. 2014, 10, 989. 23956038 10.1002/smll.201301366

[20] Y. Xu , A. Ali , K. Shehzad , N. Meng , M. Xu , Y. Zhang , X. Wang , C. Jin , H. Wang , Y. Guo , Adv. Mater. Technol. 2017, 2, 1600241.

[21] D. Estrada , Z. Li , G.‐M. Choi , S. N. Dunham , A. Serov , J. Lee , Y. Meng , F. Lian , N. C. Wang , A. Perez , npj 2D Mater. Appl. 2019, 3, 10.

[22] a) S. Shin , H. N. Cho , B. S. Kim , H. H. Cho , Thin Solid Films. 2008, 517, 933;

[23] M.‐H. Bae , Z. Li , Z. Aksamija , P. N. Martin , F. Xiong , Z.‐Y. Ong , I. Knezevic , E. Pop , Nat. Commun. 2013, 4, 1734. 23591901 10.1038/ncomms2755

[24] A. Y. Serov , Z. Y. Ong , E. Pop , Appl. Phys. Lett. 2013, 102, 3881.

[25] P. V. Bakharev , M. Huang , M. Saxena , S. W. Lee , R. S. Ruoff , Nat. Nanotechnol. 2020, 15, 59. 31819243 10.1038/s41565-019-0582-z

[26] K.‐J. Jeon , Z. Lee , E. Pollak , L. Moreschini , A. Bostwick , C.‐M. Park , R. Mendelsberg , V. Radmilovic , R. Kostecki , T. J. Richardson , ACS Nano. 2011, 5, 1042. 21204572 10.1021/nn1025274

[27] D. V. Kosynkin , A. L. Higginbotham , A. Sinitskii , J. R. Lomeda , A. Dimiev , B. K. Price , J. M. Tour , Nature. 2009, 458, 872. 19370030 10.1038/nature07872

[28] W. K. Lee , J. T. Robinson , D. Gunlycke , R. R. Stine , P. E. Sheehan , Nano Lett. 2011, 11, 5461. 22050117 10.1021/nl203225w

[29] W. Li , H. Sevinçli , G. Cuniberti , S. Roche , Phys. Rev. B. 2010, 82, 041410.

[30] F. Liu , R. Zou , N. Hu , H. Ning , C. Yan , Y. Liu , L. Wu , F. Mo , S. Fu , Nanoscale. 2019, 11, 4067. 30778431 10.1039/c8nr10468a

[31] Y. Gao , W. Yang , B. Xu , Carbon. 2016, 96, 513.

[32] W. Yao , J. Zhang , J. Ji , H. Yang , B. Zhou , X. Chen , P. Bøggild , P. U. Jepsen , J. Tang , F. Wang , Adv. Mater. 2022, 34, 2108608. 10.1002/adma.202108608 34820918

[33] X. Sun , Y. Liu , J. Shi , C. Si , J. Du , X. Liu , C. Jiang , S. Yang , Adv. Mater. 2023, 35, 2304171. 10.1002/adma.202304171 37278555

[34] S. Plimpton , J. Comput. Phys. 1995, 117, 1.

[35] J. M. Dickey , A. Paskin , Phys. Rev. 1969, 188, 1407.

