# Supplementary material for: Lateral Heterostructure Formed by Highly Thermally Conductive Fluorinated Graphene for Efficient Device Thermal Management
Source: Adv Sci (Weinh). 2024 Apr 26;11(25):2401586. doi: 10.1002/advs.202401586 (PMC11220650; doi:10.1002/advs.202401586)
Supplement: Supplementary file 1 — Supporting Information [file ADVS-11-2401586-s001.pdf]

ADVANCED SCIENCE
Open Access

Supporting Information

for *Adv. Sci.*, DOI 10.1002/advs.202401586

Lateral Heterostructure Formed by Highly Thermally Conductive Fluorinated Graphene for Efficient Device Thermal Management

Fanfan Wang, Zexin Liu, Jinfeng Li, Jian Huang, Li Fang, Xiaofeng Wang, Ruiwen Dai, Kangyong Li, Rong Zhang, Xiaoran Yang, Yue Yue, Zhiqiang Wang, Yuan Gao, Kai Yang, Lifu Zhang* and Guoqing Xin**

Supporting Information

Lateral Heterostructure Formed by Highly Thermally Conductive Fluorinated Graphene for Efficient Device Thermal Management

Fanfan Wang, Zexin Liu, Jinfeng Li, Jian Huang, Li Fang, Xiaofeng Wang, Ruiwen Dai, Kangyong Li, Rong Zhang, Xiaoran Yang, Yue Yue, Zhiqiang Wang, Yuan Gao, Kai Yang, * *Lifu Zhang,* * *and Guoqing Xin* *

Supplementary Note 1: 3D Finite Element and Uncertainty Analysis

A) 3D finite element model

To extract the thermal properties of our fluorinated graphene and the supporting Si_3N_4 substrate, we employed COMSOL software to construct a 3D finite element model of the suspended thermometry platform, as illustrated in Figure S9a. To perform the simulation, we set the bottom and side surfaces of the silicon substrate as isothermal boundary conditions, i.e., they are kept at ambient temperature, while the rest of the outer surfaces of the entire structure are treated as thermally insulated. Afterwards, a constant power was applied within the central heater to simulate the Joule heating, with a typical length of 200 μm. The temperature distribution in the steady state can be obtained by stationary calculation. To accurately calculate the thermal conductance, we observed that as heat flows from the heater towards the left and right sides, a certain portion of applied power will be lost along the heater at the edges of the suspended membrane, as depicted in Figure S9b. To estimate the percentage of heat loss (β), we extracted the temperature distribution of the cutting edge of the window on the platform. We derived the β value by calculating the heat loss to the Si substrate through the edge opens. The majority of heat was found to be lost through the electrode and the finite element analysis yielded an estimated β value of approximately 3.6%, so to simplify the calculations, we used the constant β = 3.6% in the analytical model.

B) Uncertainty Analysis

To analyze the uncertainty of our thermal conductivity measurement, we employed the classical partial derivative method:

$$\frac{u_k}{k} = \sqrt{\sum_i (S_i \times \frac{u_{x_i}}{x_i})^2} \tag{S1}$$

where u_k is the total uncertainty in the extracted thermal conductivity, u_{xi} represents the uncertainty in the i-th input parameter x_i, and the dimensionless sensitivity S_i is defined as:

$$s_i = \frac{x_i}{k}\frac{\partial k}{\partial x_i} = \frac{\partial(\ln k)}{\partial(\ln x_i)} \tag{S2}$$

To emphasize the relative importance of each input variable, we define their absolute contributions as $c_i = |s_i| \times (u_{xi}/x_i)$. For our analytical model, the main sources of uncertainty in the thermal measurement are related to the temperature measurement uncertainty ($P_H/\triangle T_H$) and the uncertainty in the geometrical dimensions (L). Therefore, S_i should be calculated based on these parameters. The uncertainty contributions of these two parameters are estimated to be 11.1% and 1.8%, respectively. Importantly, we also considered the effect of thermal contact resistance

between the fluorinated graphene and the metal heater, which is typically reported to be around 10^{-8} m^2 K W^{-1}.[1] The contact area between them is A_c = $3\times10^{-6}\times200\times10^{-6}$ = 6×10^{-10} m^2, so the thermal contact resistance per unit area can be calculated as R_c = $10^{-8}/6\times10^{-10}$ = 1.7×10 K W^{-1}. In comparison, the thermal resistance per unit area for the fluorinated graphene is R_{FG} = 1.14×10^6 K W^{-1}, which is five orders of magnitude greater than the contact thermal resistance R_c. Thus, the impact of the thermal contact resistance between fluorinated graphene and center heater can be neglected. Taking into account various sources of uncertainty, the final uncertainty for the thermal conductivity measurement of monolayer fluorinated graphene was estimated to be 12.9%, within the standard deviation of the sample.

Supplementary Note 2: Thermal finite element simulation analysis of lateral heat dissipation

To quantitatively analyze the impact of lateral heat dissipation on the overall thermal conduction of the device and the resulting current-carrying limits, we performed finite element simulations to study the thermal distribution of heterojunction devices with different graphene channel widths. As depicted in Figure S11a for the FG/Gr lateral heterojunction device under Joule heating, the temperature of the graphene strip increases. Partial Joule heat has been dissipated toward the underneath supporting substrate, while the rest has been transferred laterally into the FG on both sides. Therefore, from a power dissipation perspective, we investigated the energy of lateral heat dissipation (P') as a percentage of the total power dissipation (P_T) in devices with various channel widths as follows:

$$\frac{P'}{P_T} = \frac{2GAR\int \Delta T(x)dx}{U^2 L} \tag{S3}$$

where G represents the interfacial thermal conductance between graphene and FG, $\Delta T(x)$ is the temperature difference at the interface along the channel length (L), U and R denote the applied voltage and channel resistance (here $R = \rho L/S$), and A is the cross-sectional area perpendicular to the direction of heat flow. We first estimated the temperature difference at the interface by extracting temperature profiles on the left and right sides of the heterostructure interface (Figure S11b). Then lateral heat dissipation, P' has been obtained by the integration following Fourier's law. The research results (Figure S11c) indicate that as the graphene channel narrows, the proportion of lateral heat diffusion in the overall thermal conduction of the device increases significantly, emphasizing the importance of lateral heat spreading in narrower graphene devices, which leads to higher current density and power-handling capabilities of the transistor.

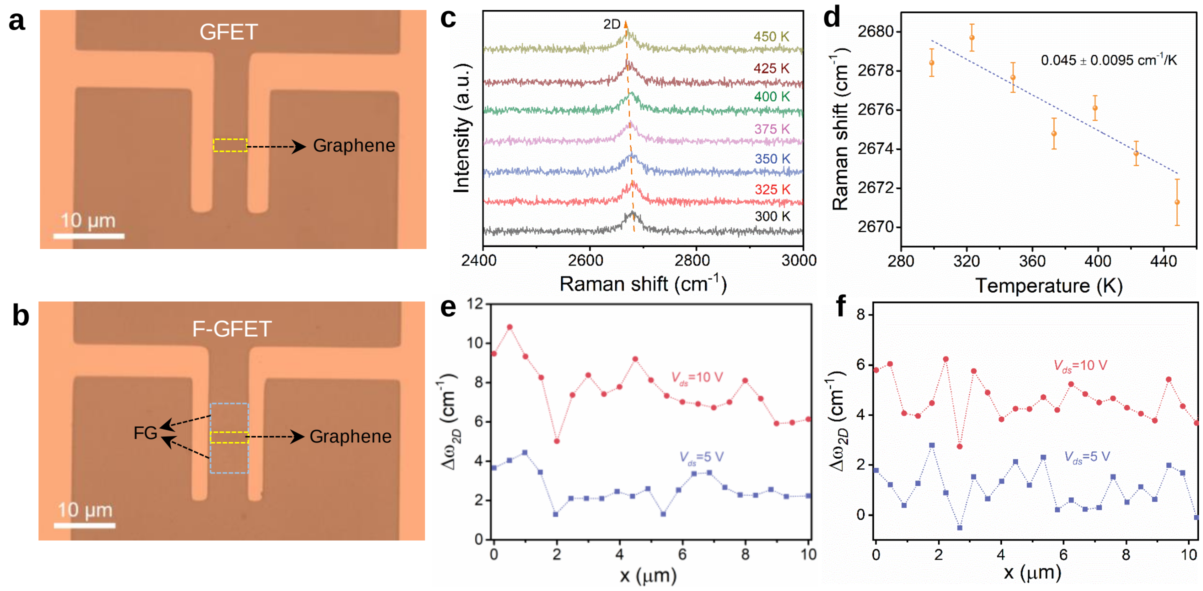

Figure S1. Position-dependent Raman thermometry measurement. a,b) Optical microscope images of GFET (a) and F-GFET (b), respectively. c) Raman spectra of monolayer graphene at different temperatures. d) Temperature dependence of the 2D band, which has been applied to obtain the Raman shift coefficient. e,f) The shift of the Raman 2D peak varies with the channel position of the GFET (e) and the F-GFET (f). The peak shift has been applied to obtain the channel temperature profile.

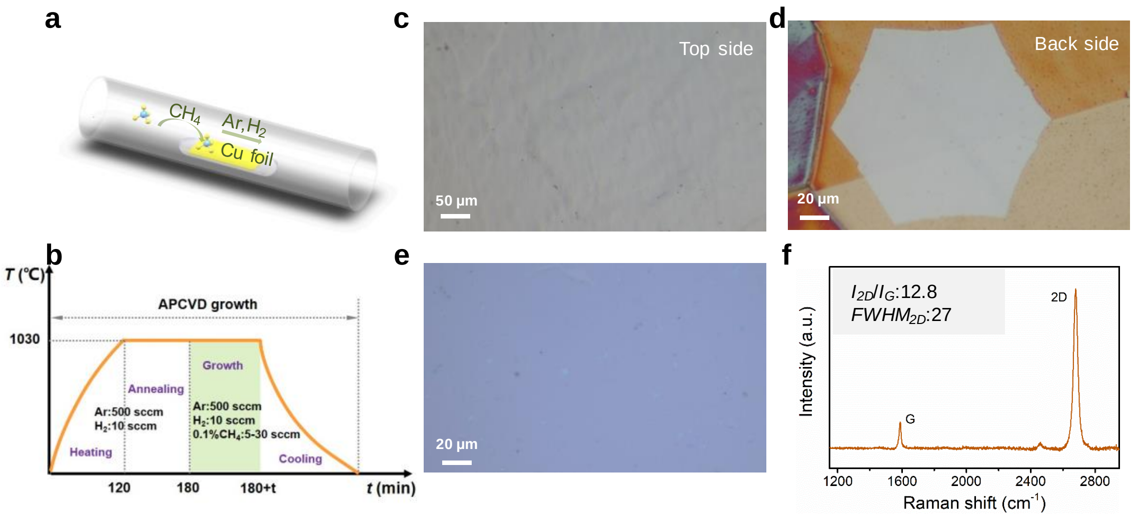

Figure S2. Growth and characterization of large-area monolayer graphene. a) Schematic of the growth of monolayer graphene on Cu foil by APCVD. b) Temperature-time profile of CVD-grown graphene. c,d) Optical microscope images of fully covered graphene (c) and graphene domain (d) grown on Cu film. e,f) Optical microscope image of as-grown sample transferred to SiO_2/Si (e), and the corresponding representative Raman spectrum (f).

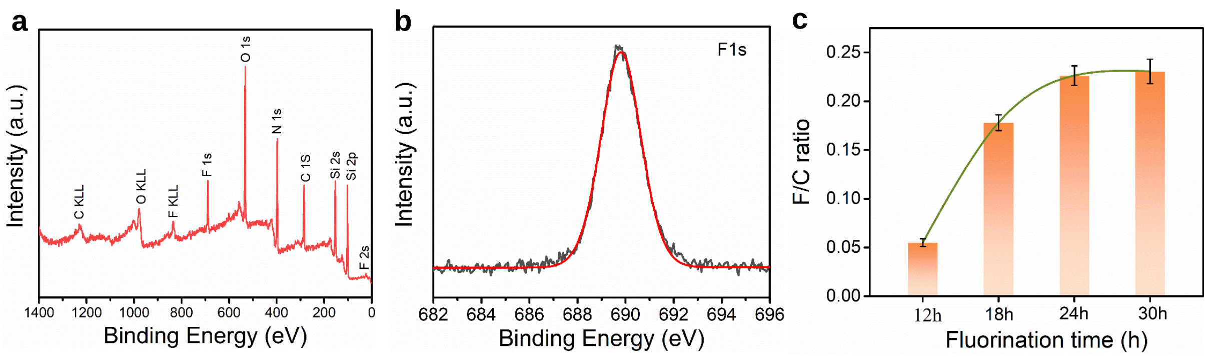

Figure S3. XPS analysis of FG. a, b) The survey XPS spectrum (a) and the fluorine peak (b) after 24 h of exposure to XeF_2. c) The correlation of fluorine to carbon atom ratios (F/C) with exposure time. The F/C ratio of the samples stabilized after 24 h, indicating that saturated fluorination of single-sided FG sample has been formed.

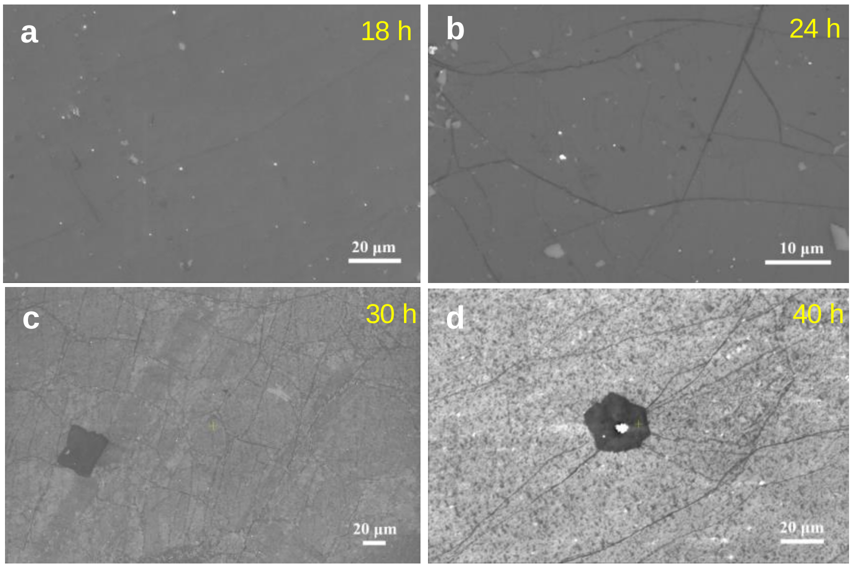

Figure S4. SEM images of FG after reaction times of 18 h (a), 24 h (b), 30 h (c), and 40 h (d). With the increase of fluorination time up to 30 h (c), the cracks on the surface of the sample increase noticeably, and the sample after 40 h (d) of reaction exhibits severe damage.

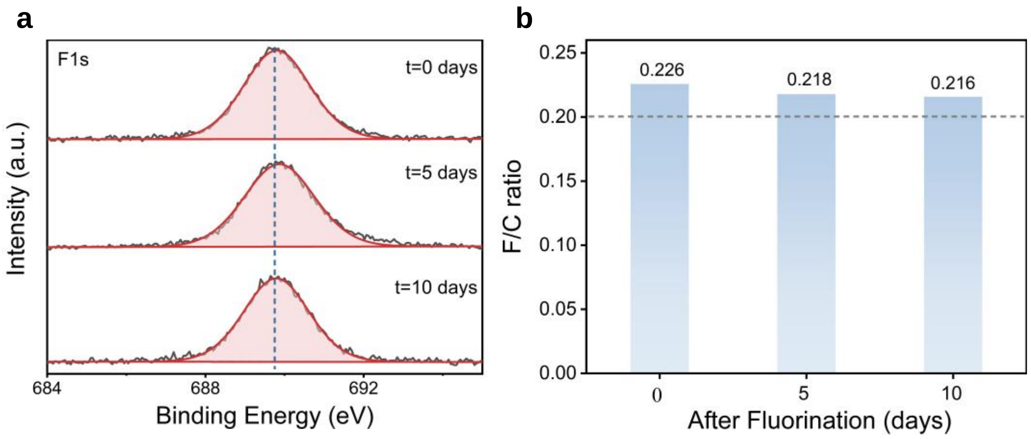

Figure S5. a) Representative XPS scans of the F 1s peak and b) the fluorine content of the FG-24 h sample under ambient storage at 0, 5 and 10 days. It can be seen that the F 1s peak position does not change significantly, and the fluorine content decreases slightly in the first few days (only 3%) and then stabilizes, suggesting that the long-term stability of the high-fluorinated samples in the atmosphere.

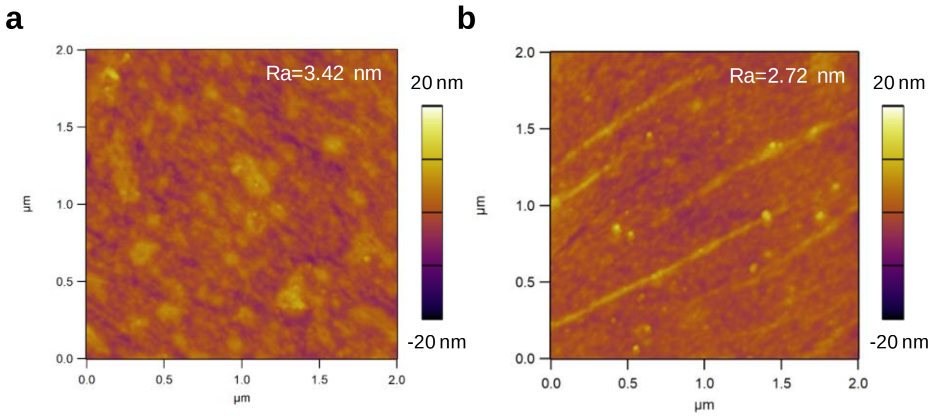

Figure S6. AFM analysis of FG. a) The sample was fluorinated directly without pre-annealing treatment. b) The fluorinated sample after annealing at 300 °C under pure Ar gas for 2 h.

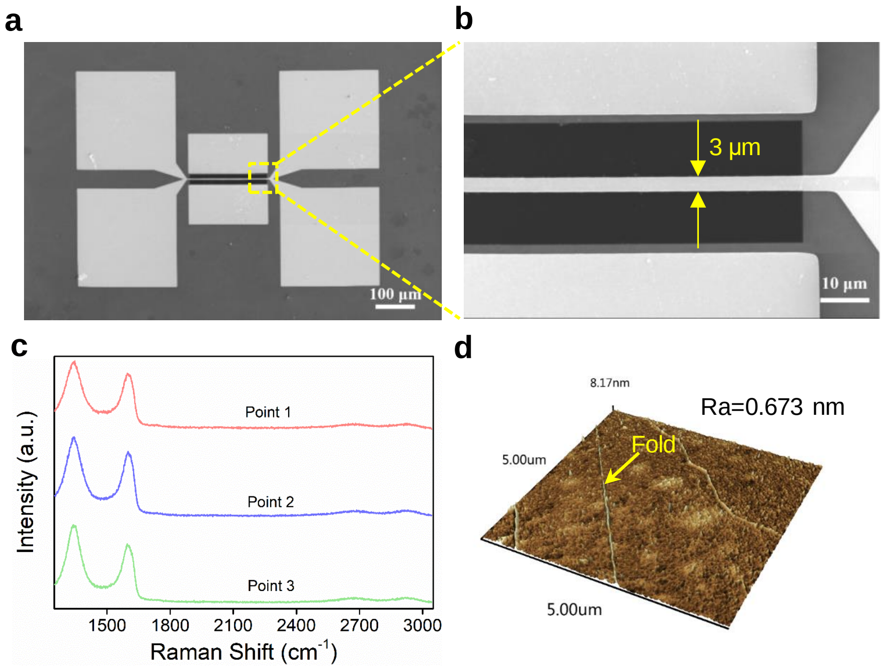

Figure S7. Characterization of electrical thermometry sample. a) SEM image of suspended thermometry platform with monolayer FG fluorinated by 24 h. b) Zoom-in of the highlighted region in (a). c) Typical Raman spectrum of monolayer FG measured at randomly different positions on the window after device preparation was completed, indicating a full coverage. d) AFM image of FG sample on the window of the device, showing a smooth surface.

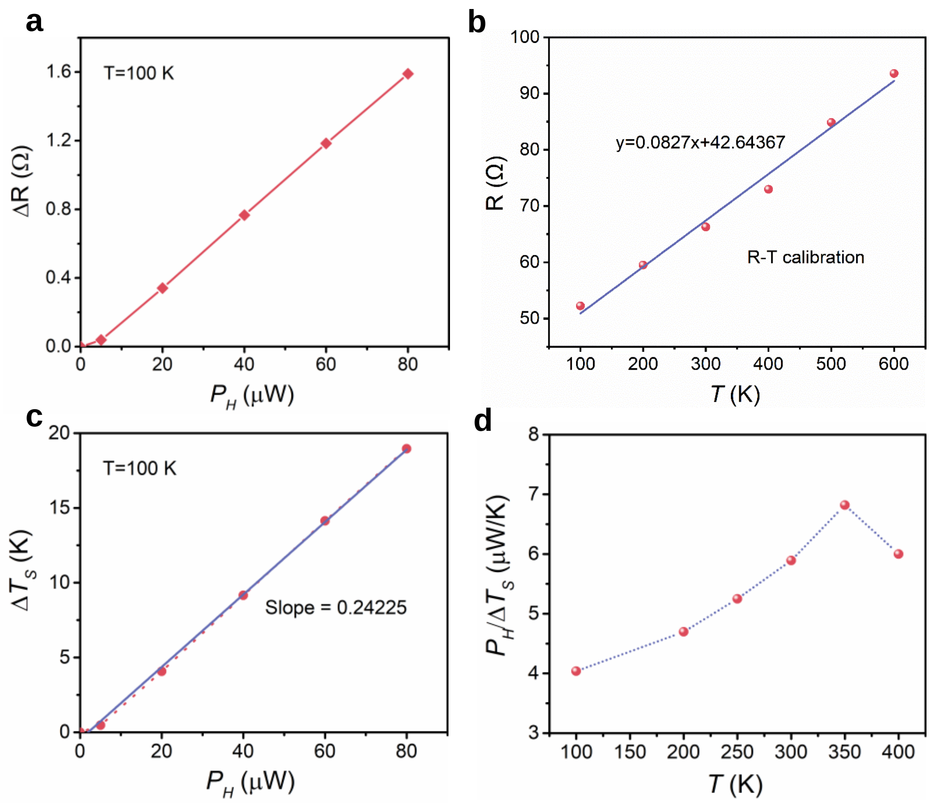

Figure S8. Thermal conductivity measurement. a) Measured resistance change as a function of heater power at T=100 K for the FG fluorinated by 24 h. b) Calibrated temperature-dependent resistance curve of the heater (or sensor). c) Converted sensor temperature rise as a function of heater power based on R-T calibration in (b). The fitted slope is $\triangle T_S/P_H$ = 0.24225 K/μW, which has been subsequently used to extract the thermal properties of the sample. d) Measured ratio of heater power to the temperature rise for FG fluorinated by 24 h.

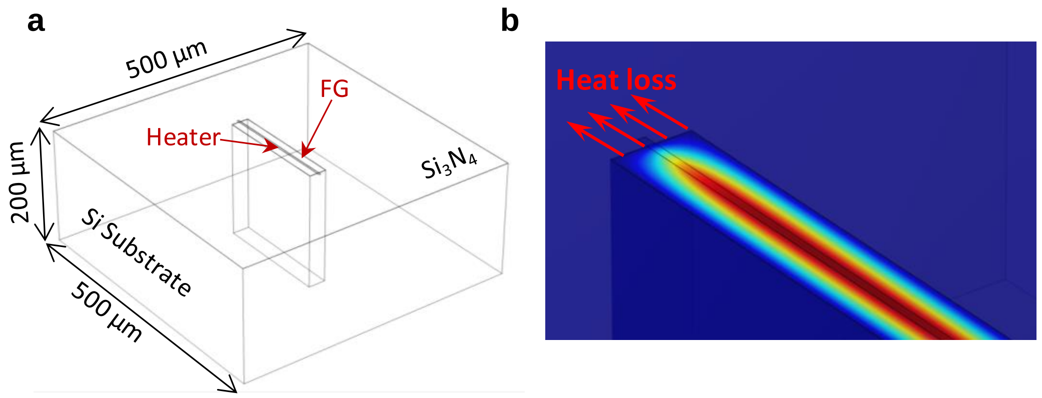

Figure S9. Heat flow analysis by finite elemental modeling (FEM). a) A typical 3D FEM model. b) Zoomed-in structure to highlight the heat loss along the heater at the edges of the suspended membrane in simulations.

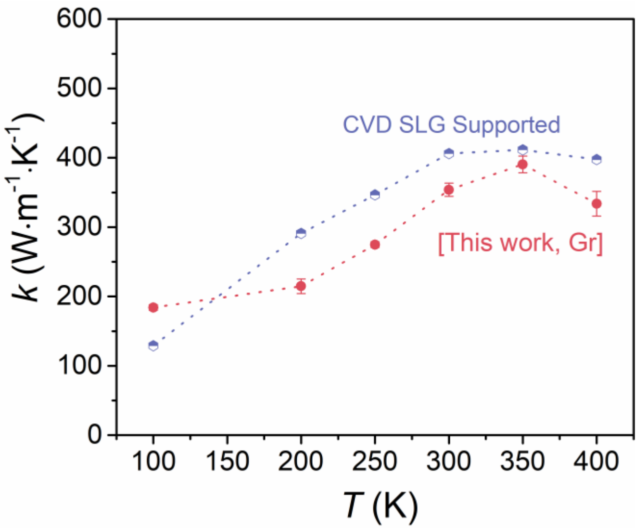

Figure S10. Thermal conductivities of monolayer graphene measured by the suspended thermometry platform. The comparison shows the reported thermal conductivities of supported single-layer graphene, [2] matching well with our data.

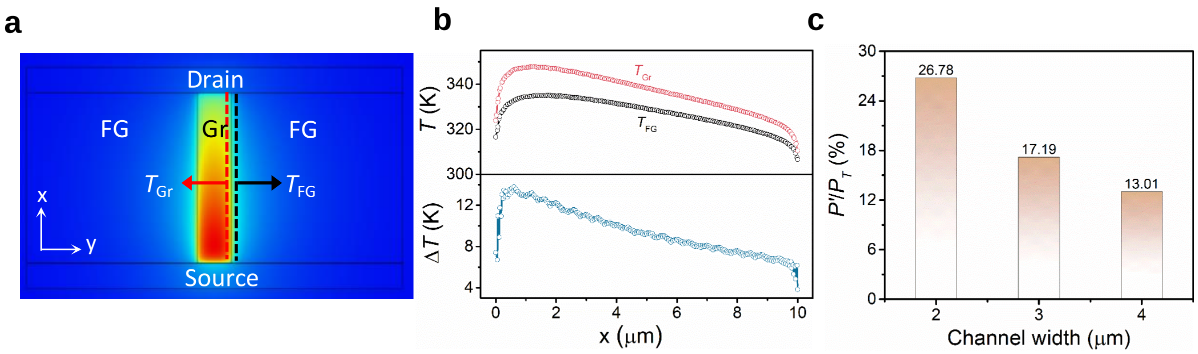

Figure S11. Analysis of lateral heat dissipation in heterostructure devices. a) Temperature distribution in a finite element simulation of the typical F-GFET lateral heterostructure device. b) The top panel shows the temperature profiles along the red and black lines in (a), representing the temperature on the cutting edge of graphene and FG along the interface, and the bottom panel shows the temperature difference curve along the heterostructure interface. c) The percentage of lateral heat dissipation from graphene to FG over the total heating power as a function of channel width.

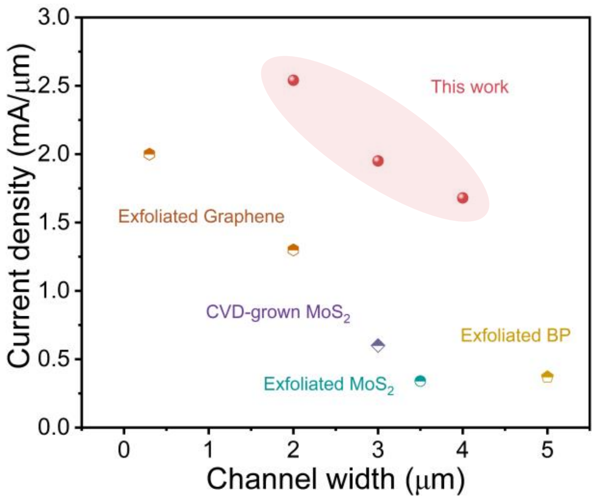

Figure S12. Comparison of maximum current density between F-GFET and literature reported devices.[3] The comparison shows that our devices with graphene/FG lateral heterostructure are much superior to the previously reported studies, which can be attributed to the improved heat dissipation performance by the heterostructure.

References

[1] a)H. Han, Y. Zhang, N. Wang, M. K. Samani, Y. Ni, Z. Y. Mijbil, M. Edwards, S. Xiong, K. Sääskilahti, M. Murugesan, *Nat. Commun.* **2016**, 7, 11281; b)R. Mao, B. D. Kong, C. Gong, S. Xu, T. Jayasekera, K. Cho, K. W. Kim, *Phys. Rev. B* **2013**, 87, 176.

[2] D. Estrada, Z. Li, G.-M. Choi, S. N. Dunham, A. Serov, J. Lee, Y. Meng, F. Lian, N. C. Wang, A. Perez, *npj 2D Mater. Appl.* **2019**, 3, 10.

[3] a)A. D. Liao, J. Z. Wu, X. Wang, K. Tahy, D. Jena, H. Dai, E. Pop, *Phys. Rev. Lett.* **2011**, 106 (25), 256801; b)X. Zhang, Q. Liao, Z. Kang, B. Liu, X. Liu, Y. Ou, J. Xiao, J. Du, Y. Liu, L. J. A. M. Gao, *Adv. Mater.* **2021**, 33 (7), 2007051; c)R. Yang, Z. Wang, P. X.-L. J. N. Feng, *Nanoscale* **2014**, 6 (21), 12383; d)F. Ali, F. Ahmed, Z. Yang, I. Moon, M. Lee, Y. Hassan, C. Lee, W. J. Yoo, *Adv. Mater. Interfaces* **2019**, 6 (2), 1801528.
